# Cancer stem cells CD133 inhibition and cytotoxicity of certain 3-phenylthiazolo[3,2-*a*]benzimidazoles: design, direct synthesis, crystal study and *in vitro* biological evaluation

**DOI:** 10.1080/14756366.2017.1347166

**Published:** 2017-07-20

**Authors:** Ghada H. Al-Ansary, Wagdy M. Eldehna, Hazem A. Ghabbour, Sara T. A. Al-Rashood, Khalid A. Al-Rashood, Radwa A. Eladwy, Abdullah Al-Dhfyan, Maha M. Kabil, Hatem A. Abdel-Aziz

**Affiliations:** a Department of Pharmaceutical Chemistry, Faculty of Pharmacy, Ain Shams University, Abbassia, Cairo, Egypt;; b Department of Pharmaceutical Chemistry, Faculty of Pharmacy, Kafrelsheikh University, Kafr El-Sheikh, Egypt;; c Department of Pharmaceutical Chemistry, College of Pharmacy, King Saud University, Riyadh, Saudi Arabia;; d Department of Medicinal Chemistry, Faculty of Pharmacy, Mansoura University, Mansoura, Egypt;; e Department of Pharmacology and Toxicology, Faculty of Pharmacy, Egyptian Russian University, Badr City, Cairo, Egypt;; f Stem Cell & Tissue Re-Engineering Program, Research Center, King Faisal Specialized Hospital & Research Center, MBC-03, Riyadh, Saudi Arabia;; g Department of Infection Control, King Saud University Medical City, Riyadh, Saudi Arabia;; h Department of Applied Organic Chemistry, National Research Center, Dokki, Giza, Egypt

**Keywords:** Cancer stem cells, CD133, cytotoxicity, colon cancer, benzimidazoles, X-ray

## Abstract

Cancer stem cells (CSCs) have been objects of intensive study since their identification in 1994. Adopting a structural rigidification approach, a novel series of 3-phenylthiazolo[3,2-*a*]benzimidazoles **4a–d** was designed and synthesised, in an attempt to develop potent anticancer agent that can target the bulk of tumour cells and CSCs. The anti-proliferative activity of the synthesised compounds was evaluated against two cell lines, namely; colon cancer HT-29 and triple negative breast cancer MDA-MB-468 cell lines. Also, their inhibitory activity against the cell surface expression of CD133 was examined. In particular, compound **4b** emerged as a promising hit molecule as it manifested good antineoplastic potency against both tested cell lines (IC_50_ = 9 and 12 μM, respectively), beside its ability to inhibit the cell surface expression of CD133 by 50% suggesting a promising potential of effectively controlling the tumour by eradicating the tumour bulk and inhibiting the proliferation of the CSCs. Moreover, compounds **4a** and **4c** showed moderate activity against HT-29 (IC_50_ = 21 and 29 μM, respectively) and MDA-MB-468 (IC_50_ = 23 and 24 μM, respectively) cell lines, while they inhibited the CD133 expression by 14% and 48%, respectively. Finally, a single crystal X-ray diffraction was recorded for compound **4d**.

## Introduction

Cancer stem cells (CSCs) paradigm spurted over the past few decades as an answering solution for the ambiguity of haematological malignancies as well as solid tumours regarding intra-tumoural heterogeneity and tumour dormancy^1^ ^,^ ^2^. Moreover, the observation that tumour has the proclivity to resist chemotherapy and even radiotherapy^3^, besides metastatic relapse that can occur more than a decade post initial treatment and clinical cure^4^ ^,^ ^5^, suggests a more circuitous aetiology for the malignancies. This surveillance devoted the scientists to abandon the postulation that tumour is a mass of homogeneous cancer cell population but rather to adopt the concept that malignancy is conceived as a “disorganised tissue”, having the CSCs at the top of the hierarchy of heterogeneous tumour tissues^6–8^.

Avalanche of scientific research postulated two key hypothetical explanations for the existence of CSCs^8^ ^,^ ^9^. CSCs may arise from normal stem cells *via* gene mutation rendering the stem cells neoplastic, or alternatively their origin might be genetic alterations of the differentiated tumour cells that ultimately acquire CSC-like features^9^.

In this view, CSCs are beheld as a distinct subpopulation of tumour cells that exhibit exclusive characteristics. Indeed, three main key properties of CSCs render them highly distinguishable^10^, (1) differentiation, as they are capable to give rise to a hierarchy of progenitor and aberrantly differentiated cells, (2) self-renewal capacity, which conserves an intact stem cell pool, (3) homeostatic control that guarantees a balance between differentiation and self-renewal in response to environmental stimuli^10^. Moreover, cunningly, the CSCs mimic their normal counterparts as they possess slow rate of proliferation rendering them resistant to conventional treatment^10^ ^,^ ^11^. Accordingly, CSCs are regarded as the main culprit that fuels tumour development, progression, metastasis, and relapse. In this insight, targeting CSCs that are the “beating heart” of the tumour is a judicious goal for establishing a platform of effective cancer therapy^4^ ^,^ ^5^ ^,^ ^11^.

Pertaining to their close similarity to normal cells, it is problematic to segregate CSCs from non-CSCs within a tumour. But for the presence of surface cell antigens, the identification and separation of tumour initiating cells from more differentiated tumour cells would not have been possible^12^. Five surface antigens whose expression is thought to indicate stem cell like properties namely, CD133, CD44, CD24, CDCP1, and CXCR4 proved to be useful for the identification and characterisation of CSCs within a tumour^12^. CD133 (Prominin-1 or AC133) is a transmembrane pentaspan protein antigen^13^ found on stem-like cells of various tissues and cancers, like brain^14^, colon^15^, breast^16^, liver^17^, pancreas^18^, kidney^19^, lung^20^, endometrium^21^, ovary^22^, and bone^23^. The supporting evidence that CD133 (+) cells had the ability to maintain survival, recurrence, metastasis, and chemotherapy resistance of neoplasms further proved that CD133 is a useful CSC marker^24^. Accordingly, it is thought to be a predictive indicator for neoplasm identification. Targeting CD133 might be a successful strategy for combating cancer.

**Figure 1.**
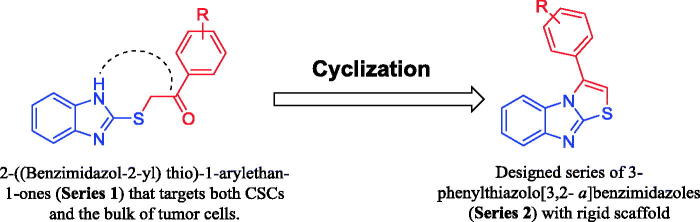
Judicious design of target 3-phenylthiazolo[3,2-*a*]benzimidazoles (**Series 2**) based on cyclisation of disclosed compounds 2-((benimidazol-2-yl)thio)-1-arylethan-1-ones (**Series 1**).

In our previous work, we synthesised a series of 2-((benzimidazol-2-yl) thio)-1-arylethan-1-ones (Series 1, Figure 1) that proved to possess good anti-proliferative activity toward HT-29 colon cancer cell line besides its capability to inhibit cell surface expression of CD133 in HT-29 cancer cells^2^. Inspired by these findings and as a part of our ongoing efforts towards developing potent anticancer agents^25^, we designed a new series of 3-phenylthiazolo[3,2-*a*]benzimidazoles (Series 2, Figure 1) based on a benzimidazole scaffold that proved to be affirmative for the anti-proliferative activity beside the CD133 inhibitory potential.

Our judicious design aimed at improving the potency of the disclosed compounds by increasing the selectivity of the synthesised compounds. This was achieved through limiting the free rotation around the single bonds in the thioethanone linker by incorporating the linker in a cyclised thiazole ring. This intervention afforded compounds that are frozen into a rigid structure thus exhibiting less isomers which augments their selectivity at the target proteins. Moreover, our design was based on previous SAR findings that highlighted the substitution of the pendent phenyl ring by an electron-donating group to be profitable for the inhibition of both the bulk tumour cells and the CSCs.

## Materials and methods

### Chemistry

Melting points were determined using a Gallenkamp melting point apparatus and are uncorrected. Infrared (IR) Spectra were recorded as KBr disks using the Perkin Elmer FT-IR (Fourier transform infrared) Spectrum BX apparatus. Mass spectra were measured on an Agilent Triple Quadrupole 6410 QQQ LC/MS (Liquid chromatography/Mass spectroscopy) equipped with an ESI (electrospray ionisation). NMR spectra were recorded on a Bruker NMR spectrometer. ^1^H spectrum was run at 500 MHz and 13C spectrum was run at 125 MHz in deuterated dimethyl sulfoxide (DMSO-d_6_). Chemical shifts are expressed in *δ* values (ppm) using the solvent peak as internal standard. All coupling constant (*J*) values are given in hertz. The abbreviations used are as follows: s, singlet; d, doublet; m, multiplet. Elemental analyses were carried out at the Regional Center for Mycology and Biotechnology, Al-Azhar University, Cairo, Egypt. Analytical thin layer chromatography (TLC) on silica gel plates containing UV indicator was employed routinely to follow the course of reactions and to check the purity of products. All reagents and solvents were purified and dried by standard techniques.

#### General procedure for synthesis of sulphate salts 3a–d

To a solution of the appropriate acetophenone **1a–d** (5 mmol) in glacial acetic acid (10 ml), 2-mercaptobenzimidazole **2** (0.75 g, 5 mmol) and conc. sulphuric acid (50 mmol) were added. The reaction mixture was heated under reflux for 2 h. The solid product obtained upon cooling was filtered off, washed with cold water then with petroleum ether, and recrystallised from ethanol to afford the corresponding sulphate salts **3a–d**, respectively.

#### General procedure for preparation of 3-(3-anyl)benzo[4,5]imidazo[2,1-b]thiazoles 4a–d

To a suspension of the appropriate sulphate salts **3a–d** (2 mmol) in water (10 ml), an aqueous solution of sodium bicarbonate was added. The reaction mixture was stirred at room temperature for 2 h. The solid formed was collected by filtration, washed with water, dried, and crystallised from ethanol to afford compounds **4a–d**, respectively.

##### 3-(3-Methoxyphenyl)benzo[4,5]imidazo[2,1-b]thiazole (4a)

White crystals (yield 80%), m.p. 173–175 °C; ^1^H NMR (DMSO-d_6_) *δ* ppm: 3.84 (s, 3H, OCH_3_), 7.13 (d, 1H, Ar–H, *J* = 7.5 Hz), 7.20–7.32 (m, 6H, Ar–H), 7.54 (t, 1H, Ar–H, *J* = 8.0 Hz), 7.71 (d, 1H, Ar–H, *J* = 8.0 Hz); 13C NMR (DMSO-d_6_) *δ* ppm: 55.86 (OCH_3_), 109.23, 112.01, 114.68, 116.44, 119.23, 120.86, 121.47, 123.61, 130.61, 130.69, 133.66, 148.64, 157.03, 159.92; Anal. Calcd. for C_16_H_12_N_2_OS: C, 68.55; H, 4.31; N, 9.99; Found C, 68.73; H, 4.28; N, 10.12.

##### 4-(Benzo[4,5]imidazo[2,1-b]thiazol-3-yl)-2-methoxyphenol (4b)

White crystals (yield 78%), m.p. 190–193 °C; ^1^H NMR (DMSO-d_6_) *δ* ppm: 3.82 (s, 3H, OCH_3_), 6.99 (d, 1H, Ar–H, *J* = 8.0 Hz), 7.09 (s, 1H, Ar–H), 7.12 (t, 2H, Ar–H, *J* = 7.5 Hz), 7.27–7.31 (m, 3H, Ar–H), 7.69 (d, 1H, Ar–H, *J* = 8.0 Hz), 9.67 (s, 1H, OH, D_2_O exchangeable); 13C NMR (DMSO-d_6_) *δ* ppm: 56.23 (OCH_3_), 107.63, 112.07, 113.40, 116.14, 119.11, 120.06, 120.72, 122.38, 123.48, 130.22, 134.22, 148.17, 148.67, 148.77, 156.94; Anal. Calcd. for C_16_H_12_N_2_O_2_S: C, 64.85; H, 4.08; N, 9.45; Found C, 65.14; H, 4.02; N, 9.34.

##### 3-(3,4,5-Trimethoxyphenyl)benzo[4,5]imidazo[2,1-b]thiazole (4c)

White crystals (yield 83%), m.p. 177–179 °C; ^1^H NMR (DMSO-d_6_) *δ* ppm: 3.79 (s, 6H, 2 OCH_3_), 3.83 (s, 3H, OCH_3_), 7.07 (s, 2H, Ar–H), 7.22–7.38 (m, 4H, Ar–H), 7.71 (d, 1H, Ar–H, *J* = 8.0 Hz); 13C NMR (DMSO-d_6_) *δ* ppm: 56.62 (OCH_3_), 60.71 (OCH_3_), 106.95, 108.79, 112.23, 119.19, 120.89, 123.58, 124.64, 130.21, 133.83, 139.20, 148.68, 153.59, 156.93; Anal. Calcd. for C_18_H_16_N_2_O_3_S: C, 63.51; H, 4.74; N, 8.23; Found C, 63.69; H, 4.69; N, 8.11.

##### 4-(Benzo[4,5]imidazo[2,1-b]thiazol-3-yl)aniline (4d)

White crystals (yield 75%), m.p. 185–186 °C; ^1^H NMR (DMSO-d_6_) *δ* ppm: 5.28 (s, 2H, NH_2_), 6.73 (d, 2H, Ar–H, *J* = 8.0 Hz), 7.22–7.38 (m, 4H, Ar–H), 7.59 (d, 2H, Ar–H, *J* = 8.0 Hz), 7.68 (d, 1H, Ar–H, *J* = 8.0 Hz); Anal. Calcd. for C_15_H_11_N_3_S: C, 67.90; H, 4.18; N, 15.84; Found C, 68.15; H, 4.14; N, 15.73.

### X-ray crystallographic analysis

The measurements of the crystal of compound **4d** were performed on a Bruker SMART APEX II D8 Venture diffractometer with graphite-monochromated Mo *Kα* radiation (*λ* = 0.71073 Å) at 100 K. The structure was solved by direct method and refined with SHELXTL. E-maps provided the positions of all the non-H-atoms. The full-matrix least-squares refinement was carried out on *F* ^2^’s using anisotropic temperature factors for all non-*H*-atoms. Crystallographic data for the structure reported in this paper have been deposited at the Cambridge Crystallographic Data Centre and allocated with the deposition number: CCDC 1429525.

### Biological evaluations

#### *In vitro* anti-proliferative activity

Anti-proliferative activity of the synthesised 3-phenylthiazolo[3,2-*a*]benzimidazoles **4a–d** was evaluated at Stem Cell Therapy and Tissue Reengineering Program, King Faisal Specialized Hospital and Research Center, Riyadh, Saudi Arabia. *In vitro* anti-proliferative activity was measured by the cell growth inhibition assay. This assay was conducted by use WST-1(water soluble tetrazolium-1) reagent^26^ for determination of IC_50_ for each compound. HT-29 colon cancer cell line and MDA-MB-468 triple negative breast cancer cell line were purchased from the American Type Culture Collection. Cells were maintained in RPMI 1640 (Sigma-Aldrich, St. Louis, MO), supplemented with 10% FBS (Fetal Bovine Serum) (Lonza Group, Basel, Switzerland), 100 IU/mL penicillin, 100 mg/mL streptomycin, and 2 mmol/L L-glutamine (Sigma). Cells were seeded into 96-well plates at 0.4 * 10^4^/well and incubated overnight. The medium was replaced with fresh one containing the desired concentrations of the test compounds. After 48 h, 10 μl of the WST-1 reagent were added to each well and the plates were re-incubated for 4 h at 37 °C. The amount of formazan was quantified using ELISA (Enzyme Linked Immunosorbent Assay) reader at 450 nm.

#### CD133 expression measure by flow cytometry

HT-29 and MDA-MB-468 cells harvested, washed, and then the cells were stained with conjugated monoclonal antibodies CD133-APC (Miltenyi Biotec, Bergisch Gladbach, Germany). The analyses were performed on a BD LSR II™ (BD Biosciences, San Jose, CA). Debris and cell clusters were excluded during side-scatter and forward-scatter analyses.

## Results and discussion

### Chemistry

**Scheme 1.**
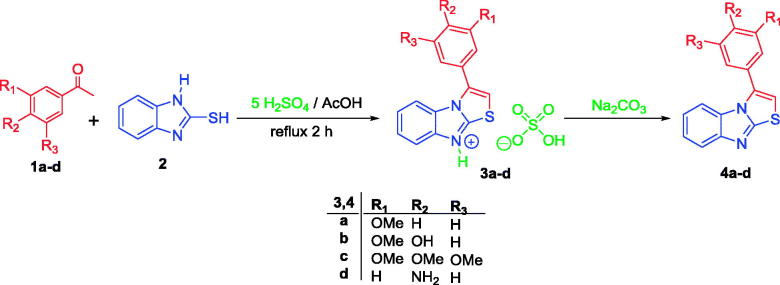
Synthesis of target 3-phenylthiazolo[3,2-*a*]benzimidazoles **4a–d**.

In the present work, target compounds **4a–d** were prepared according to Scheme 1. In a one-pot two-components heterocyclisation process, sulphate salts **3a–d** were obtained *via* the reaction of acetophenone **1a–d** with 2-mercaptobenzimidazole **2** in refluxing acetic acid in the presence of five equivalents of sulphuric acid. Next, neutralisation of such sulphate salts **3a–d** was carried out through stirring with aqueous solution of sodium bicarbonate to furnish the 3-phenylthiazolo[3,2-*a*]benzimidazoles **4a–d**, with 75–83% yield (Scheme 1). All spectral and elemental analyses were consistent with the proposed structures of the prepared compounds.

### X-ray crystallographic study for 4d

**Figure 2.**
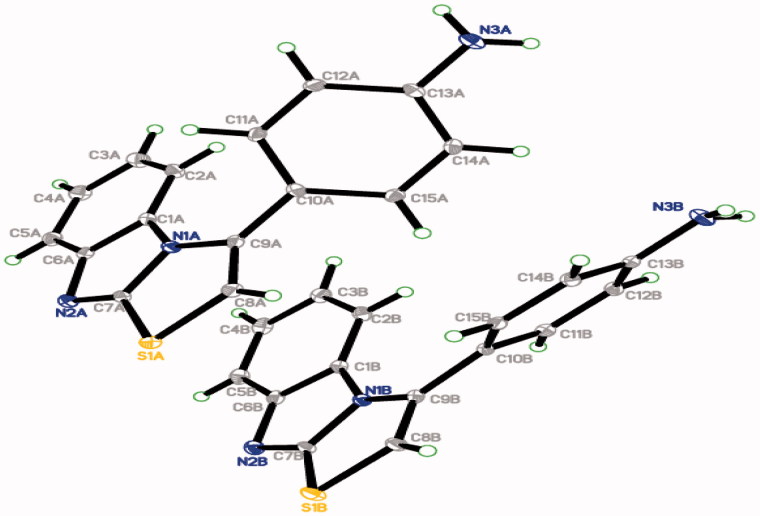
An ORTEP diagram of final X-ray structure of compound **4d**.

Characterisation of compound **4d** was conducted by a single crystal X-ray structural analysis. The structure was solved with direct method and refined by SHELXTL^27^. Crystallographic data of compound **4d** is deposited with the Cambridge Crystallographic Data Centre with deposition number CCDC 1429525. The crystallographic structure of **4d** is represented in Figure 2. The single crystal X-ray study for such compound unambiguously defines its exact structure. Crystal packing of **4d** showed the intermolecular hydrogen bonds N3A—H1NA···N2A and N3B—H1NB···N2B (Supplementary data). The crystallographic data and hydrogen-bond geometry of **4d** are presented in Tables 1 and 2, respectively, while selected geometric parameters are illustrated in the Supplementary data.

**Table 1.** Crystallographic data and refinements for compound **4d**.

Crystal data	
C_15_H_11_N_3_S	*V* = 2399.62 (14) Å^3^
*M_*r*_* = 265.33	*Z* = 8
Monoclinic, **P**2_1_/**n**	Mo *K*α radiation
*a* = 9.6379 (3) Å	μ = 0.26 mm^−1^
*b* = 22.8517 (8) Å	*T* = 100 K
*c* = 10.9316 (4) Å	0.42 × 0.14 × 0.12 mm
β = 94.666 (1)°	*F*(000) = 1104
*Data collection*	
Bruker APEX-II D8 venture diffractometer	*R*_int_ = 0.041
20125 measured reflections	*θ*_max_ = 27.5°
5502 independent reflections	4463 reflections with *I* > 2σ(*I*)
*Refinement*	
*R*[*F*^2^ > 2*σ*(*F*^2^)] = 0.041	0 restraints
*wR*(*F*^2^) = 0.112	H atoms treated by a mixture of independent and constrained refinement
*S* = 1.04	*w* = 1/[*σ*^2^(*F*_o_^2^) + (0.0579*P*)^2^ + 1.2502*P*]
where *P* = (*F*_o_^2^ + 2*F*_c_^2^)/3	
5502 reflections	Δ*ρ*_max_ = 0.41 e Å^−3^
359 parameters	Δ*ρ*_min_ = −0.31 e Å^−3^

**Table 2.** Hydrogen-bond geometry (Å, °) of **4d**.

*D*—H···*A*	*D*—H	*H···A*	*D*···*A*	*D*—H···*A*
N3A—H1NA···N2A^i^	0.91 (3)	2.19 (2)	3.095 (2)	168 (2)
N3B—H1NB···N2B^i^	0.93 (2)	2.18 (2)	3.046 (2)	155 (2)

Symmetry codes: (i) *x*, *y*, *z* − 1.

### Biological evaluation

Anti-proliferative activity of the prepared 3-phenylthiazolo[3,2-*a*]benzimidazoles **4a–d** was evaluated against human colon cancer cell line HT-29 and triple negative breast cancer cell line MDA-MB-468 using the WST-1 assay as described by Ngamwongsatit et al^26^. 5-Fluorouracil was used as a positive control for its well-known clinical utility for managing malignant carcinomas. The anti-proliferative activities were expressed as growth inhibitory concentration (IC_50_) values. Among the tested compounds, compound **4b** bearing a terminal phenyl ring substituted with two electron-donating groups proved to be the most potent against both cell lines; HT-29 and MDA-MB-468 with IC_50_ values of 9 and 12 μM, respectively. Compared to the positive control 5-FU (IC_50_ values of 15 and 41 μM, respectively), these results proclaimed compound **4b** to be superior to the reference drug. Moreover, compounds **4a** and **4c** showed moderate activity against HT-29 cell line with IC_50_ values of 21 and 29 μM, respectively, which are comparable to that of 5-FU (IC_50_ = 15 μM). Luckily, both compounds evinced good antineoplastic potency against MDA-MB-468 cell line relative to 5-FU (IC_50_ values of 23, 24, and 41 μM, respectively). Unfortunately, compound **4d** bearing a *p*-amino phenyl group did not manifest any significant anti-proliferative activity against the cancer colon cell line HT-29 while it demonstrated moderate activity against triple negative breast cancer cell line MDA-MB-468 (53 μM) (Table 3).

**Table 3.** *In vitro* anti-proliferative activity of compounds **4a–d** against colon HT-29 and breast MDA-MB-468 cancer cell lines.


				IC_50_ (μM)^a^	
Comp.	R_1_	R_2_	R_3_	HT-29	MDA-MB-468
**4a**	OMe	H	H	21 ± 1.5	24 ± 1.7
**4b**	OMe	OH	H	10 ± 0.7	13 ± 0.9
**4c**	OMe	OMe	OMe	29 ± 2.3	24 ± 2.2
**4d**	H	NH_2_	H	NA^b^	53 ± 4.7
**5-Fluorouracil**				16 ± 1.6	41 ± 3.8

a IC_50_ values are the mean ± SD of three separate experiments.

b NA: compounds having IC_50_ value >100 μM.

Moreover, the inhibitory effect of the prepared 3-phenylthiazolo[3,2-*a*]benzimidazoles **4a–d** on cell surface expression of CD133 was evaluated at 10 μM *via* flow cytometry. The analysis was performed on a BD LSR II™ (BD Biosciences). The results expressed as a side population inhibition (%). Scrutinizing the obtained results disclosed that compound **4a** bearing a *m*-methoy phenyl group only limitedly inhibited the CD133 by 13%, whereas compound **4b** that possesses an extra electron donating group; an additional *p*-hydroxy group significantly inhibited the CD133 expression by 50%. Coherent to these findings, compound **4c** that has three methoxy groups grafted on its terminal phenyl ring produced a comparable inhibition of CD133 (48%). These findings are in accordance with the previous conclusion that outlined the importance of incorporation of a lipophilic electron-donating group represented by a terminal phenyl ring substituted with a methoxy or a hydroxyl group. Regrettably, compound **4d** containing a *p*-amino phenyl ring did not display any marked activity against the cell surface expression of CD133 (Table 4).

**Table 4.** Inhibition (%) of cell surface expression of CD133 on HT-29 cancer cells at 10 μM.

Comp.	Inhibition (%) ± SD^a^
Untreated	–
**4a**	14 ± 1.0
**4b**	50 ± 4.3
**4c**	48 ± 3.8
**4d**	NA^b^

a Values are the mean ± SD of three separate experiments.

b Compounds having inhibition % <10.

It is worth noting that the cytotoxic activities were decreased in the order of **4b > 4a > 4c > 4d**, while the order was **4b > 4c > 4a > 4d** for the inhibitory activity towards CD133 surface expression, which suggesting absence of a correlation between the two activities that may be attributable to the different phenotypic characteristics and different proliferative potentials.

## Conclusions

In conclusion, we designed and synthesised a novel series of 3-arylthiazolo[3,2-*a*]benzimidazoles **4a–d** (Series 2) based on structural rigidification of a series of 2-((benzimidazol-2-yl) thio)-1-arylethan-1-ones (Series 1) that proved to have antineoplastic activity and inhibitory activity of cell surface expression of CD133. The anti-proliferative activity of the synthesised compounds was evaluated against two cell lines; colon cancer cell line HT-29 and triple negative breast cancer cell line MDA-MB-468. Moreover, their inhibitory activity against the cell surface expression of CD133 was determined in an attempt to explore their potential to eradicate CSCs as well as the tumour bulk cells. Compound **4b** emerged as a promising hit molecule as it manifested excellent antineoplastic potency against both tested cell lines (IC_50_ values of 9 and 12 μM, respectively) beside its ability to inhibit the cell surface expression of CD133 by 50% suggesting a promising potential of effectively controlling the tumour by eradicating the tumour bulk and inhibiting the proliferation of the CSCs. Moreover, compounds **4a** and **4d** exhibited good anti-proliferative activity against both cell lines and also significant inhibition potential of cell surface expression of CD133. On the other hand, structure of compound **4d** was further substantiated *via* X-ray single crystal analysis.

## Supplementary Material

IENZ_1347166_Supplementary_Material.pdf
