# Supplementary material for: Cancer stem cells CD133 inhibition and cytotoxicity of certain 3-phenylthiazolo[3,2-a]benzimidazoles: design, direct synthesis, crystal study and in vitro biological evaluation
Source: J Enzyme Inhib Med Chem. 2017 Jul 20;32(1):986–91. doi: 10.1080/14756366.2017.1347166 (PMC6010115; doi:10.1080/14756366.2017.1347166)
Supplement: IENZ_1347166_Supplementary_Material.pdf [file IENZ_A_1347166_SM3162.pdf]

Electronic Supplementary Information

Cancer stem cells CD133 inhibition and cytotoxicity of certain 3-phenylthiazolo[3,2-*a*]benzimidazoles: design, direct synthesis, crystal study and *in vitro* biological evaluation

Ghada H. Al-Ansary[1], Wagdy M. Eldehna[2,*], Hazem A. Ghabbour[3,4], Sara T.A. Al-Rashood[3], Khalid A. Al-Rashood[3], Radwa A. Eladwy[5], Abdullah Al-Dhfyan[6], Maha M. Kabil[7], Hatem A. Abdel-Aziz[8,*]

[1]*Department of Pharmaceutical Chemistry, Faculty of Pharmacy, Ain Shams University, Abbassia, Cairo 11566, Egypt.*
[2]*Department of Pharmaceutical Chemistry, Faculty of Pharmacy, Kafrelsheikh University, Kafr El-Sheikh 33516, Egypt.*
[3]*Department of Pharmaceutical Chemistry, College of Pharmacy, King Saud University, P.O. Box 2457, Riyadh 11451, Saudi Arabia.*
[4]*Department of Medicinal Chemistry, Faculty of Pharmacy, Mansoura University, Mansoura P.O. 35516, Egypt.*
[5]*Department of Pharmacology and Toxicology, Faculty of Pharmacy, Egyptian Russian University, Badr City, Cairo 11829, Egypt*
[6]*Stem Cell & Tissue Re-Engineering Program, Research Center, King Faisal Specialized Hospital & Research Center, MBC-03, P.O. Box 3354, Riyadh 11211, Saudi Arabia*
[7]*Department of Infection Control, King Saud University Medical City, Riyadh Saudi Arabia*
[8]*Department of Applied Organic Chemistry, National Research Center, Dokki, Giza, P.O. Box 12622, Egypt*

1. X-ray crystallographic analysis

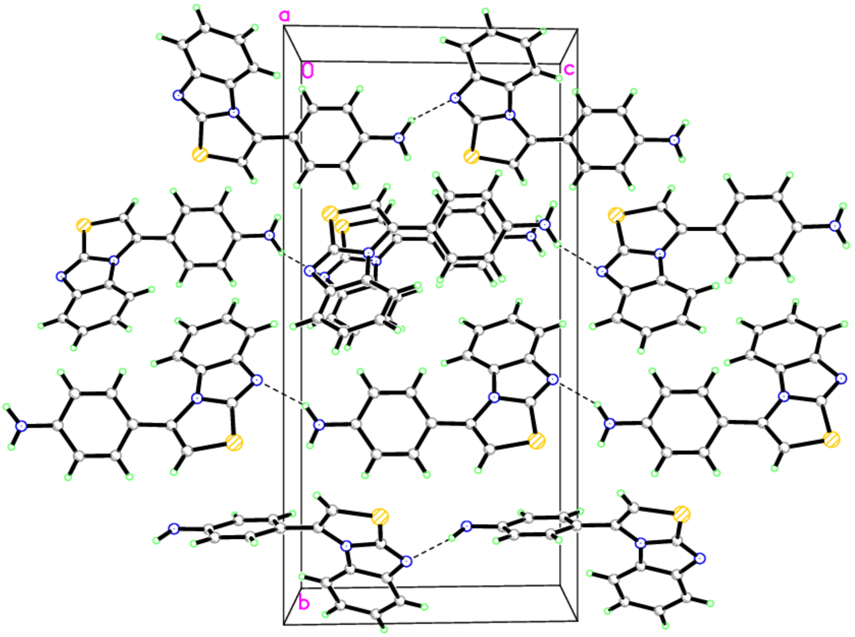

Figure 1S. Crystal packing of **4d** showing intermolecular hydrogen bonds as dashed lines.

Table 1S. Selected geometric parameters (Å, °) for compound **4d**.

**Bond distance**			
S1A—C7A	1.7288 (17)	N2A—C6A	1.399 (2)
S1A—C8A	1.7517 (17)	N3A—C13A	1.390 (2)
S1B—C7B	1.7304 (17)	N1B—C1B	1.397 (2)
S1B—C8B	1.7475 (17)	N1B—C7B	1.383 (2)
N1A—C9A	1.408 (2)	N1B—C9B	1.406 (2)
N1A—C7A	1.386 (2)	N2B—C6B	1.401 (2)
N1A—C1A	1.395 (2)	N2B—C7B	1.311 (2)
N2A—C7A	1.313 (2)	N3B—C13B	1.389 (2)
**Bond angle**			
C7A—S1A—C8A	90.00 (8)	S1A—C8A—C9A	113.81 (13)
C7B—S1B—C8B	89.57 (8)	N1A—C9A—C10A	121.36 (15)
C1A—N1A—C9A	139.57 (14)	N1A—C9A—C8A	110.76 (14)
C7A—N1A—C9A	114.48 (14)	N3A—C13A—C12A	120.59 (16)
C1A—N1A—C7A	105.70 (13)	N3A—C13A—C14A	120.78 (16)
C6A—N2A—C7A	103.03 (14)	N1B—C1B—C6B	104.51 (14)
C1B—N1B—C9B	139.31 (14)	N1B—C1B—C2B	133.09 (15)
C1B—N1B—C7B	105.53 (13)	N2B—C6B—C5B	128.29 (15)
C7B—N1B—C9B	114.35 (13)	N2B—C6B—C1B	111.56 (14)
C6B—N2B—C7B	102.92 (14)	S1B—C7B—N1B	111.26 (12)
N1A—C1A—C6A	104.42 (14)	S1B—C7B—N2B	132.91 (13)
N1A—C1A—C2A	133.52 (15)	N1B—C7B—N2B	115.43 (14)
N2A—C6A—C1A	111.65 (15)	S1B—C8B—C9B	114.28 (13)
N2A—C6A—C5A	128.36 (15)	N1B—C9B—C10B	123.11 (14)
S1A—C7A—N2A	133.89 (13)	N1B—C9B—C8B	110.53 (14)
N1A—C7A—N2A	115.14 (14)	N3B—C13B—C14B	120.33 (16)
S1A—C7A—N1A	110.94 (12)	N3B—C13B—C12B	121.44 (16)

2. Representative ^{1}H and ^{13}C NMR spectra

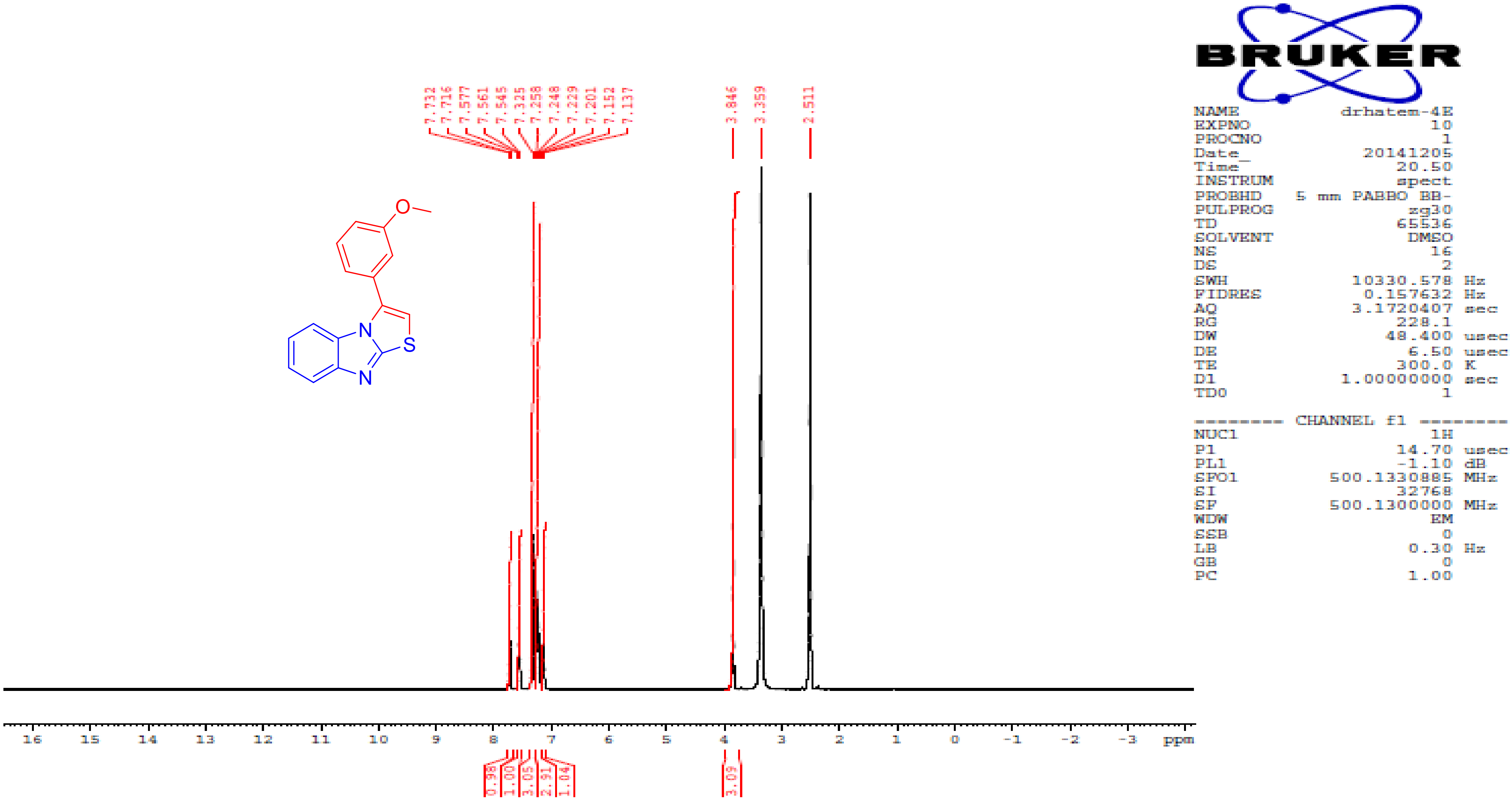

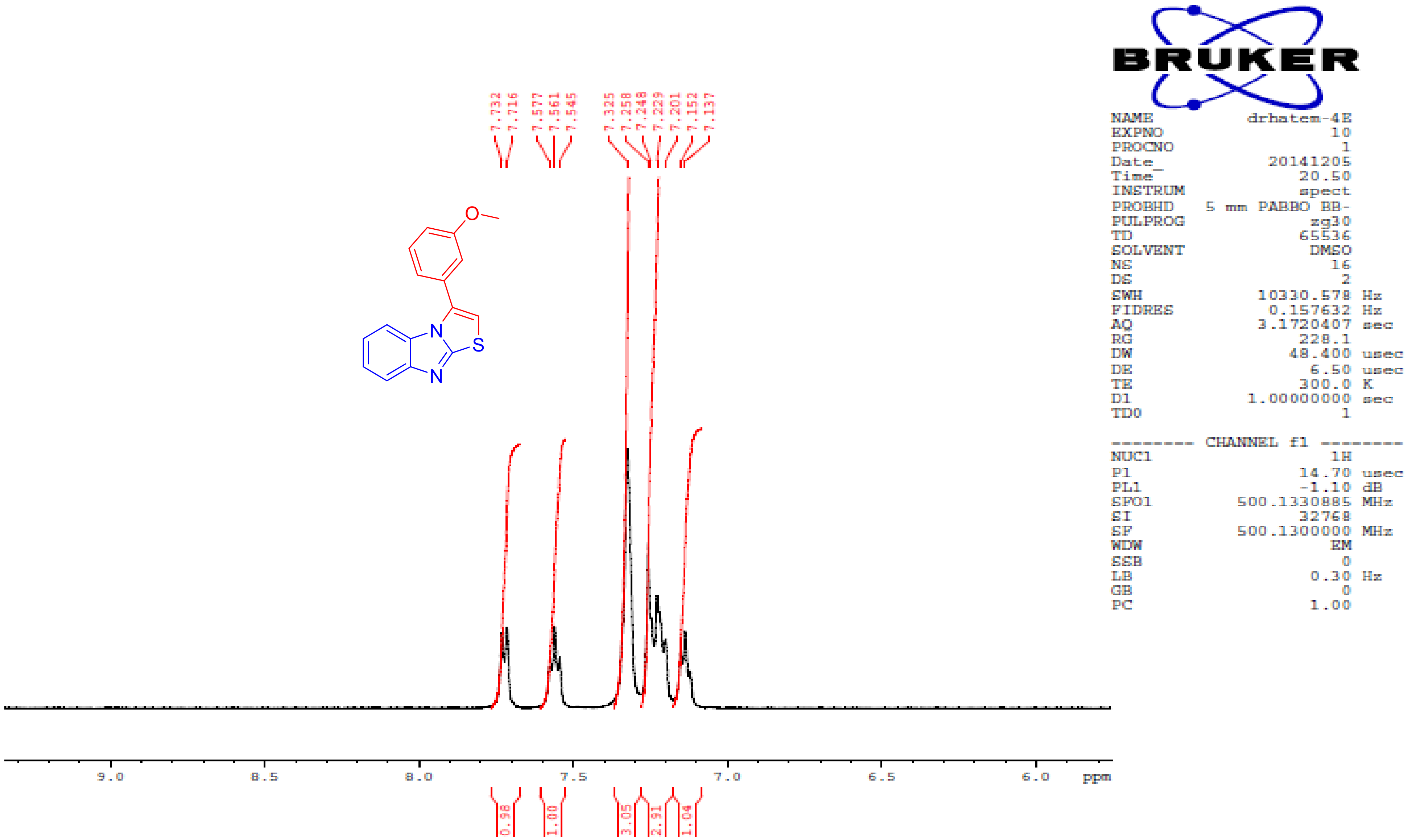

7.732
7.716
7.577
7.561
7.545
7.325
7.258
7.248
7.229
7.201
7.152
7.137
O
N
S
N
9.0
8.5
8.0
7.5
7.0
6.5
6.0
ppm
0.98
1.00
3.05
2.91
1.04
BRUKER
NAME drhatem-4E
EXPNO 10
PROCNO 1
Date_ 20141205
Time 20.50
INSTRUM spect
PROBHD 5 mm PABBO BB-
PULPROG zg30
TD 65536
SOLVENT DMSO
NS 16
DS 2
SWH 10330.578 Hz
FIDRES 0.157632 Hz
AQ 3.1720407 sec
RG 228.1
DW 48.400 usec
DE 6.50 usec
TE 300.0 K
D1 1.00000000 sec
TD0 1
======== CHANNEL f1 ========
NUC1 1H
P1 14.70 usec
PL1 -1.10 dB
SFO1 500.1330885 MHz
SI 32768
SF 500.1300000 MHz
WDW EM
SSB 0
LB 0.30 Hz
GB 0
PC 1.00

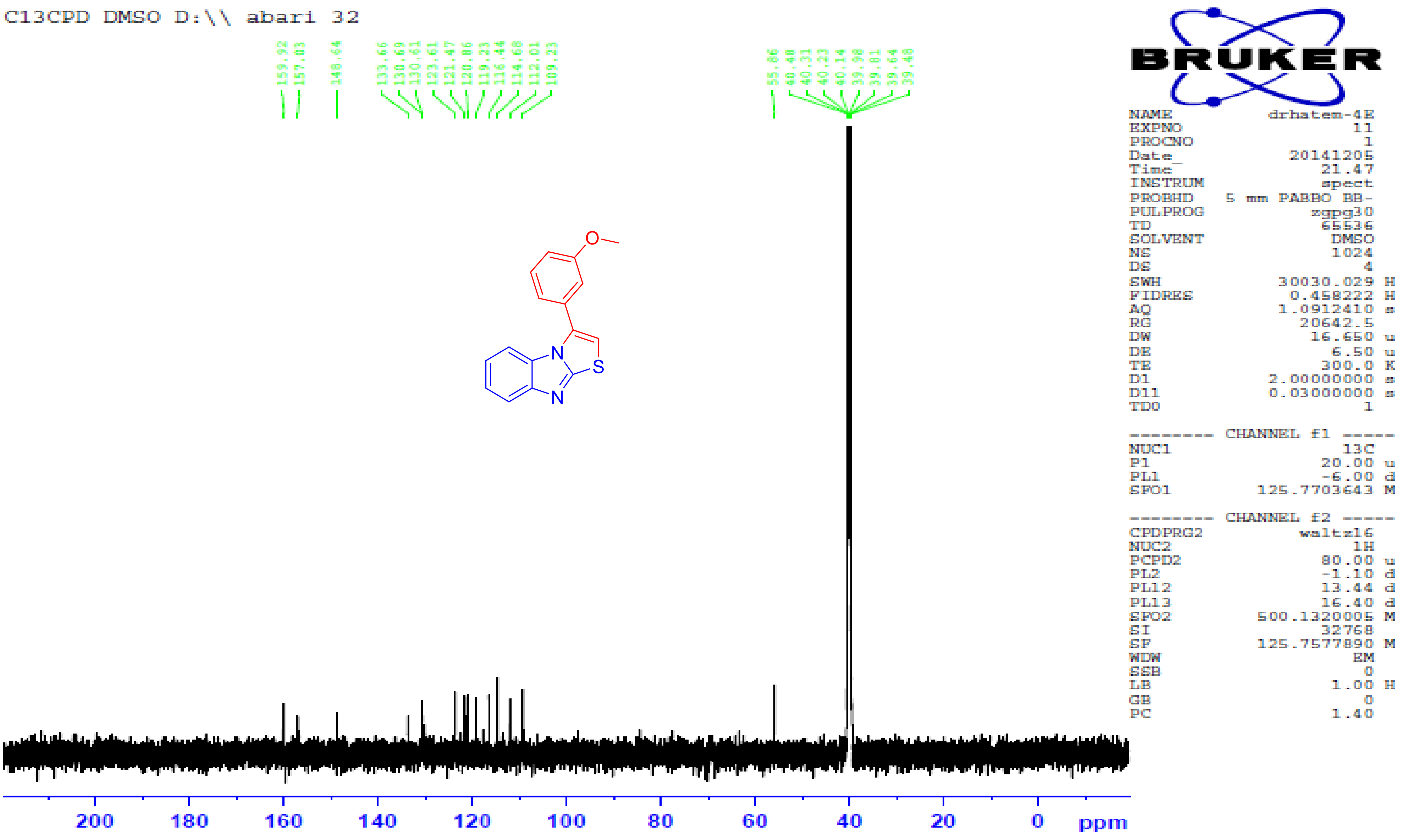
C13CPD DMSO D:\\ abari 32
159.92
157.03
148.64
133.66
130.69
130.61
123.61
121.47
120.86
119.23
116.44
114.68
112.01
109.23
55.86
40.48
40.31
40.23
40.14
39.98
39.81
39.64
39.48
O
N
S
N
BRUKER
NAME drhatem-4E
EXPNO 11
PROCNO 1
Date_ 20141205
Time 21.47
INSTRUM spect
PROBHD 5 mm PABBO BB-
PULPROG zgpg30
TD 65536
SOLVENT DMSO
NS 1024
DS 4
SWH 30030.029 H
FIDRES 0.458222 H
AQ 1.0912410 s
RG 20642.5
DW 16.650 u
DE 6.50 u
TE 300.0 K
D1 2.00000000 s
D11 0.03000000 s
TD0 1
======== CHANNEL f1 =====
NUC1 13C
P1 20.00 u
PL1 -6.00 d
SFO1 125.7703643 M
======== CHANNEL f2 =====
CPDPRG2 waltz16
NUC2 1H
PCPD2 80.00 u
PL2 -1.10 d
PL12 13.44 d
PL13 16.40 d
SFO2 500.1320005 M
SI 32768
SF 125.7577890 M
WDW EM
SSB 0
LB 1.00 H
GB 0
PC 1.40
200
180
160
140
120
100
80
60
40
20
0
ppm

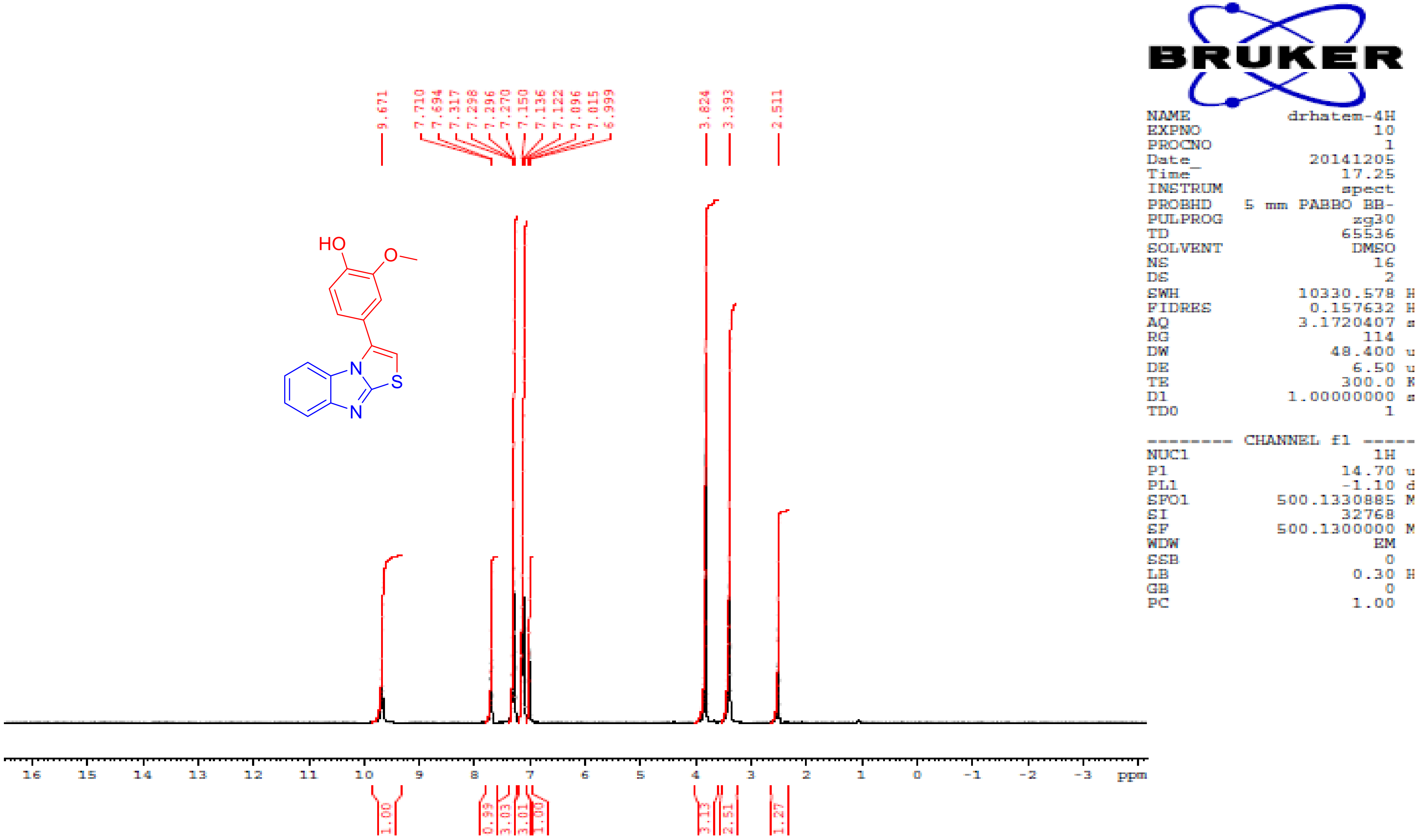

9.671
7.710
7.694
7.317
7.298
7.296
7.270
7.150
7.136
7.122
7.096
7.015
6.999
3.824
3.393
2.511
HO
O
N
S
N
BRUKER
NAME drhatem-4H
EXPNO 10
PROCNO 1
Date_ 20141205
Time 17.25
INSTRUM spect
PROBHD 5 mm PABBO BB-
PULPROG zg30
TD 65536
SOLVENT DMSO
NS 16
DS 2
SWH 10330.578 H
FIDRES 0.157632 H
AQ 3.1720407 s
RG 114
DW 48.400 u
DE 6.50 u
TE 300.0 K
D1 1.00000000 s
TD0 1
======== CHANNEL f1 =====
NUC1 1H
P1 14.70 u
PL1 -1.10 d
SFO1 500.1330885 M
SI 32768
SF 500.1300000 M
WDW EM
SSB 0
LB 0.30 H
GB 0
PC 1.00
16 15 14 13 12 11 10 9 8 7 6 5 4 3 2 1 0 -1 -2 -3 ppm
1.00
0.99
3.03
3.01
1.00
3.13
2.51
1.27

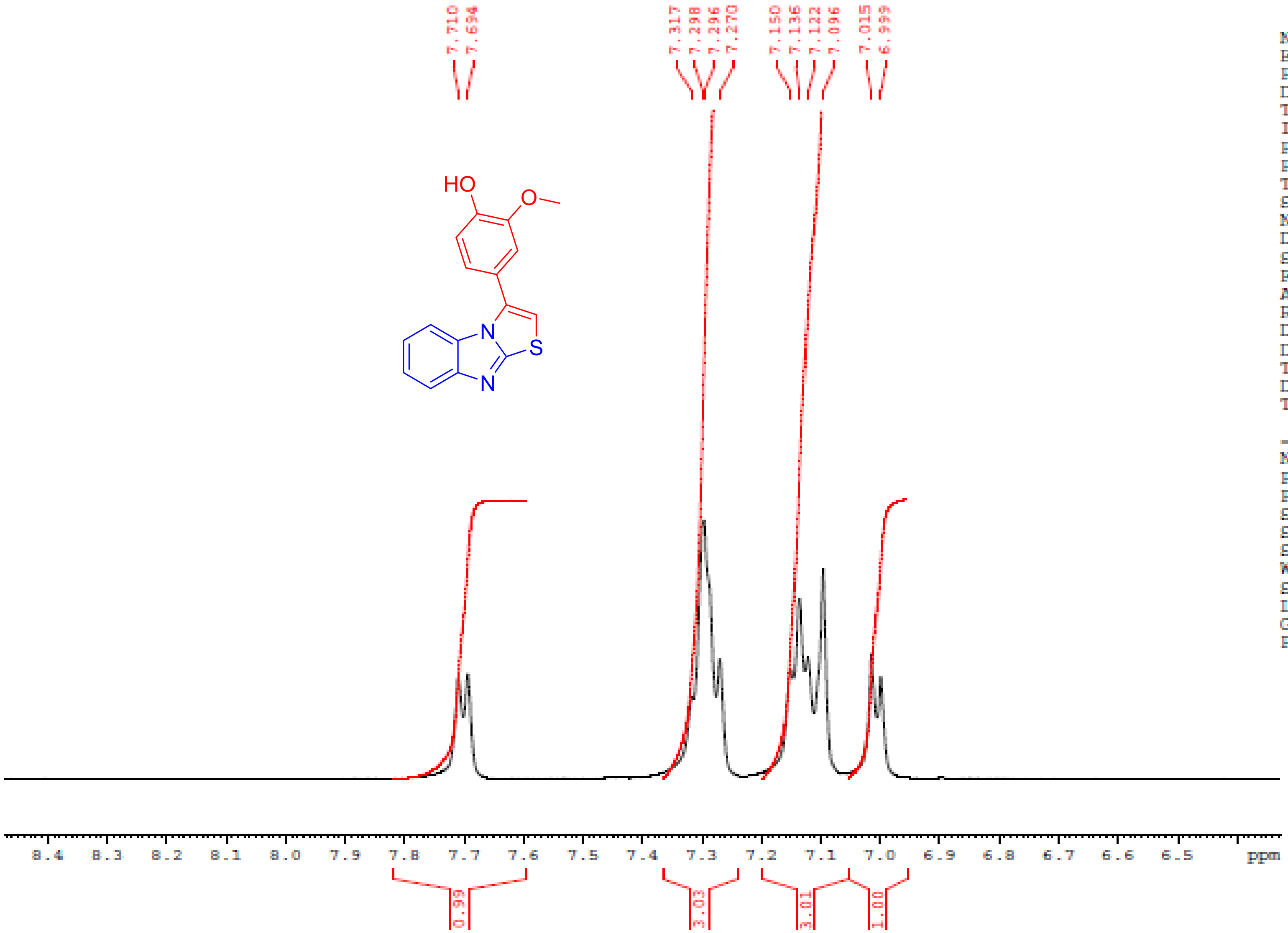

NAME	drhatem-4H
EXPNO	10
PROCNO	1
Date_	20141205
Time	17.25
INSTRUM	spect
PROBHD	5 mm PABBO BB-
PULPROG	zg30
TD	65536
SOLVENT	DMSO
NS	16
DS	2
SWH	10330.578 Hz
FIDRES	0.157632 Hz
AQ	3.1720407 se
RG	114
DW	48.400 us
DE	6.50 us
TE	300.0 K
D1	1.00000000 se
TD0	1

======== CHANNEL f1 ======

NUC1	1H
P1	14.70 us
PL1	-1.10 dB
SFO1	500.1330885 MH
SI	32768
SF	500.1300000 MH
WDW	EM
SSB	0
LB	0.30 Hz
GB	0
PC	1.00

C13CPD DMSO D:\\ abari 29

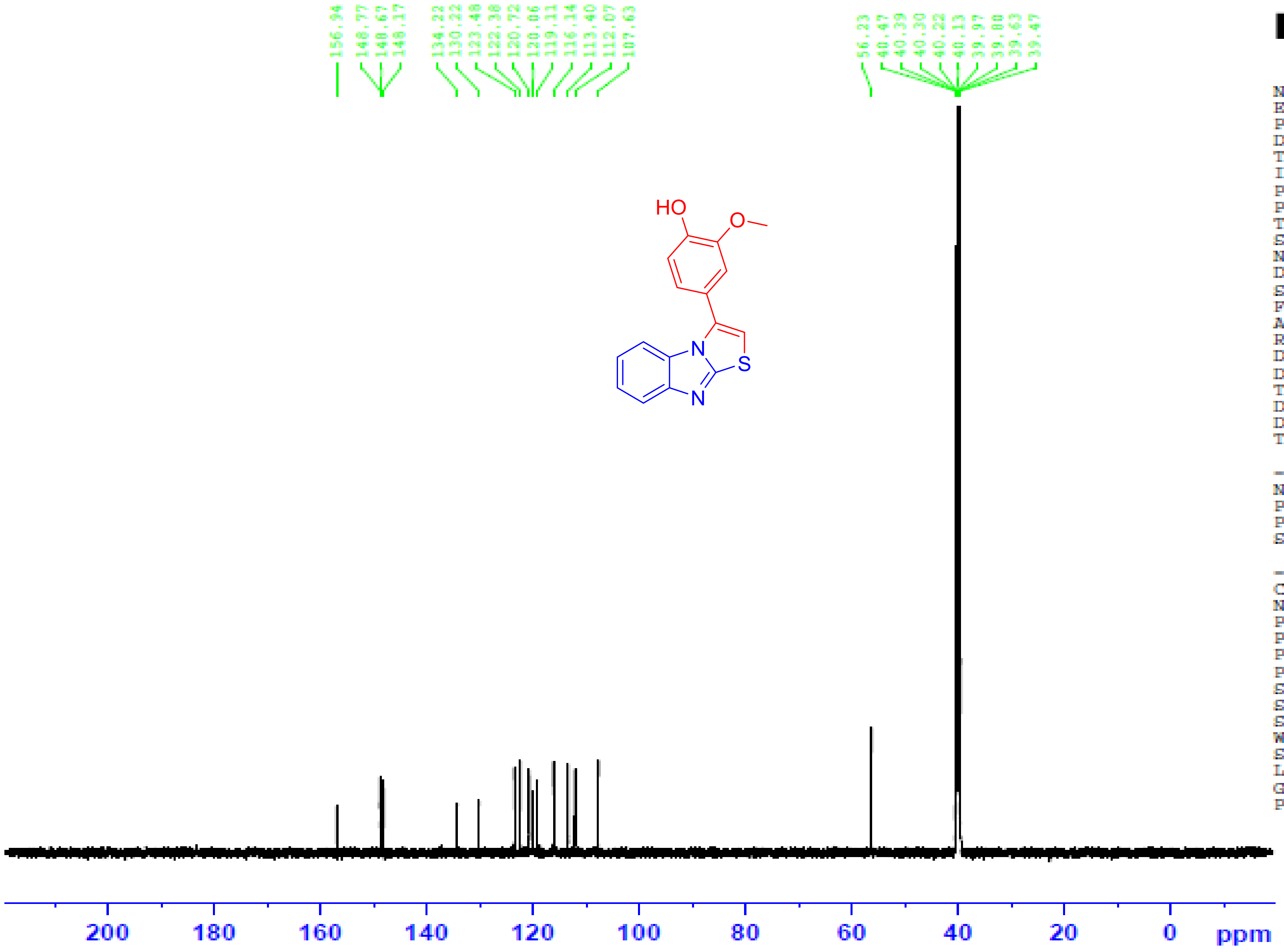

NAME	drhatem-4H
EXPNO	11
PROCNO	1
Date_	20141205
Time	18.23
INSTRUM	spect
PROBHD	5 mm PABBO BB-
PULPROG	zgpg30
TD	65536
SOLVENT	DMSO
NS	1024
DS	4
SWH	30030.029 Hz
FIDRES	0.458222 Hz
AQ	1.0912410 se
RG	14596.5
DW	16.650 us
DE	6.50 us
TE	300.0 K
D1	2.00000000 se
D11	0.03000000 se
TD0	1

======== CHANNEL f1 ======

NUC1	13C
P1	20.00 us
PL1	-6.00 dB
SFO1	125.7703643 MH

======== CHANNEL f2 ======

CPDPRG2	waltz16
NUC2	1H
PCPD2	80.00 us
PL2	-1.10 dB
PL12	13.44 dB
PL13	16.40 dB
SFO2	500.1320005 MH
SI	32768
SF	125.7577890 MH
WDW	EM
SSB	0
LB	1.00 Hz
GB	0
PC	1.40

ON DMSO D:\\ abar

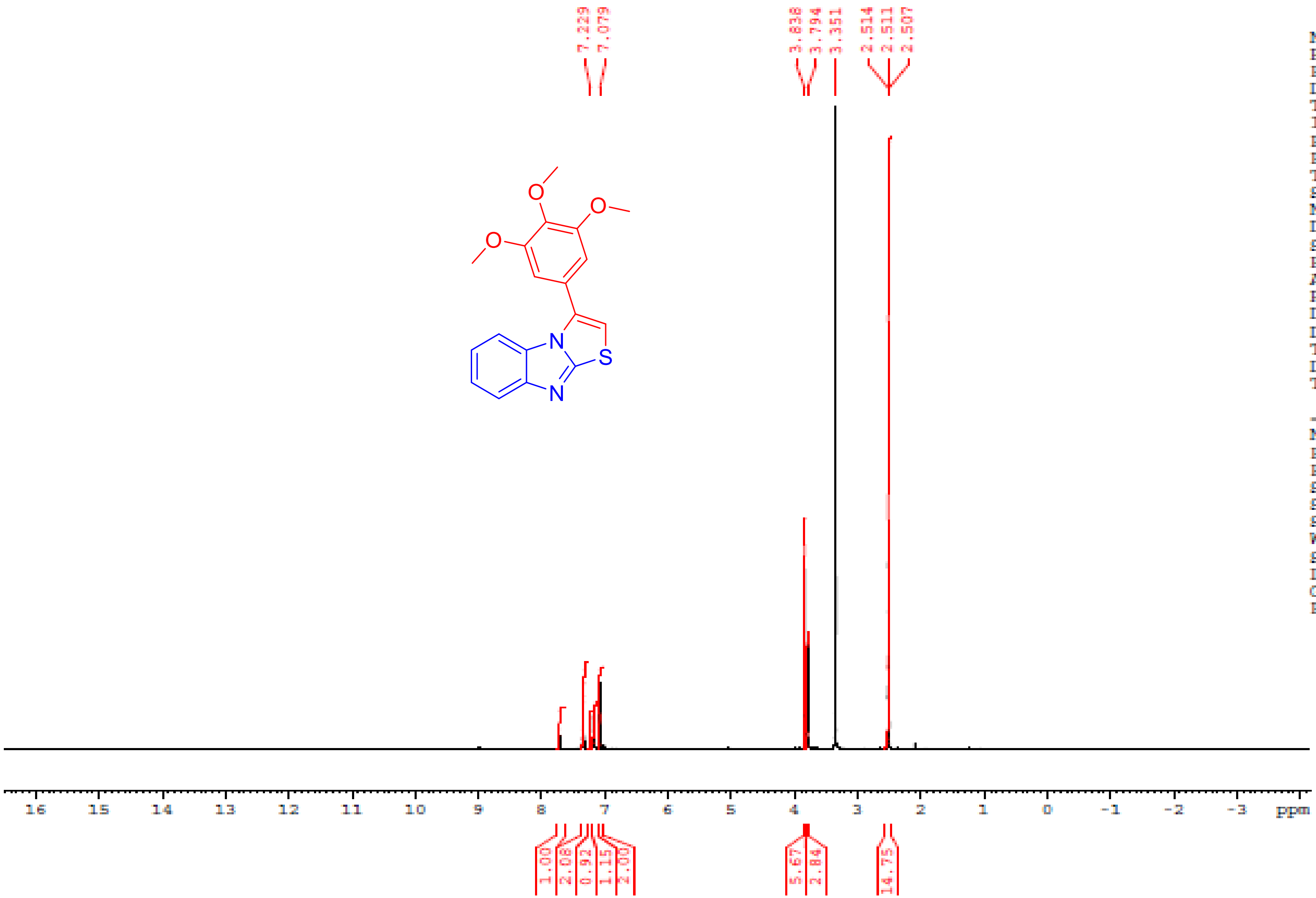

NAME	drrashood-4N
EXPNO	10
PROCNO	1
Date_	20141226
Time	6.13
INSTRUM	spect
PROBHD	5 mm PABBO BB-
PULPROG	zg30
TD	65536
SOLVENT	DMSO
NS	16
DS	2
SWH	10330.578 Hz
FIDRES	0.157632 Hz
AQ	3.1720407 sec
RG	256
DW	48.400 usec
DE	6.50 usec
TE	300.0 K
D1	1.00000000 sec
TD0	1

======== CHANNEL f1 ========

NUC1	1H
P1	14.70 usec
PL1	-1.10 dB
SFO1	500.1330885 MHz
SI	32768
SF	500.1300000 MHz
WDW	EM
SSB	0
LB	0.30 Hz
GB	0
PC	1.00

C13CPD DMSO D:\\ abari 20

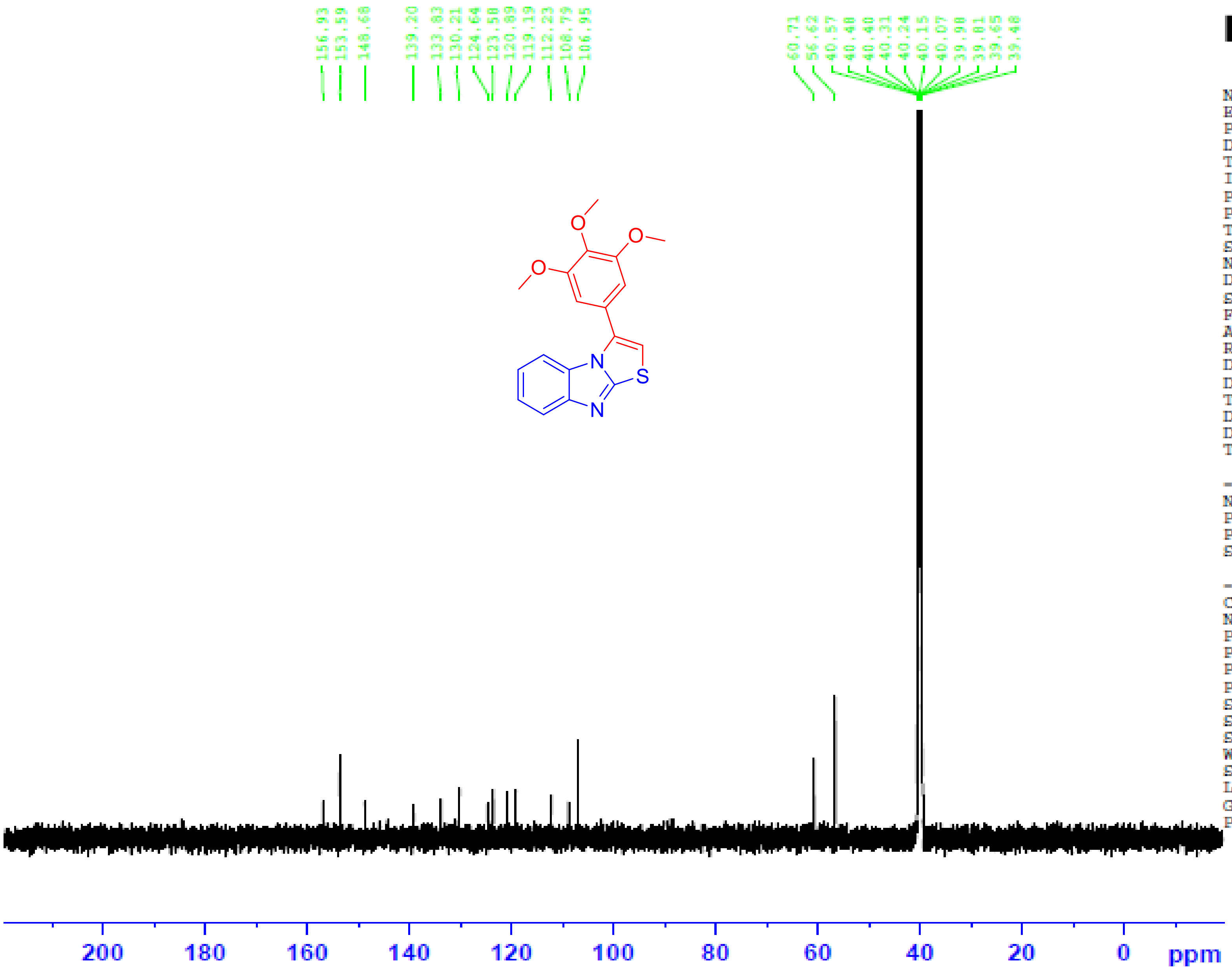

BRUKER

NAME	drrashood-4N	
EXPNO	11	
PROCNO	1	
Date_	20141226	
Time	8.53	
INSTRUM	spect	
PROBHD	5 mm PABBO BB-	
PULPROG	zgpg30	
TD	65536	
SOLVENT	DMSO	
NS	3000	
DS	4	
SWH	30030.029	Hz
FIDRES	0.458222	Hz
AQ	1.0912410	sec
RG	16384	
DW	16.650	usec
DE	6.50	usec
TE	300.0	K
D1	2.00000000	sec
D11	0.03000000	sec
TD0	1	

======== CHANNEL f1 ========

NUC1	13C	
P1	20.00	usec
PL1	-6.00	dB
SFO1	125.7703643	MHz

======== CHANNEL f2 ========

CPDPRG2	waltz16	
NUC2	1H	
PCPD2	80.00	usec
PL2	-1.10	dB
PL12	13.44	dB
PL13	16.40	dB
SFO2	500.1320005	MHz
SI	32768	
SF	125.7577890	MHz
WDW	EM	
SSB	0	
LB	1.00	Hz
GB	0	
PC	1.40	
